# Polyomavirus BK Replication in *De Novo* Kidney Transplant Patients Receiving Tacrolimus or Cyclosporine: A Prospective, Randomized, Multicenter Study

**DOI:** 10.1111/j.1600-6143.2012.04320.x

**Published:** 2012-11-08

**Authors:** H H Hirsch, F Vincenti, S Friman, M Tuncer, F Citterio, A Wiecek, E H Scheuermann, M Klinger, G Russ, M D Pescovitz, H Prestele

**Affiliations:** a Transplantation and Clinical Virology, Department Biomedicine—Building Petersplatz, University of BaselBasel, Switzerland; b Division of Infectious Diseases and Hospital Epidemiology, University Hospital BaselSwitzerland; c University of California San Francisco, Kidney Transplant ServiceSan Francisco, CA; d Department of Transplantation and Liver Surgery, Sahlgrenska University HospitalGothenburg, Sweden; e MedicalPark Hospital, Organ Transplant CenterAntalya, Turkey; f Division of Organ Transplantation, Department of Surgery, Catholic University of the Sacred HeartRome, Italy; g Department of Nephrology, Endocrinology and Metabolic Diseases, Medical University of SilesiaKatowice, Poland; h Department of Nephrology, University HospitalFrankfurt am Main, Germany; i Department of Nephrology and Transplantation Medicine, Medical UniversityWroclaw, Poland; j The Queen Elizabeth HospitalWoodwille, Australia; k Departments of Surgery and Microbiology/Immunology, Indiana UniversityIndianapolis, IN; l Novartis Pharma AGBasel, Switzerland

**Keywords:** BK virus, cyclosporine, immunosuppression, polyomavirus, risk factor, steroids, tacrolimus, transplantation

## Abstract

Polyomavirus BK (BKV)-associated nephropathy causes premature kidney transplant (KT) failure. BKV viruria and viremia are biomarkers of disease progression, but associated risk factors are controversial. A total of 682 KT patients receiving basiliximab, mycophenolic acid (MPA), corticosteroids were randomized 1:1 to cyclosporine (CsA) or tacrolimus (Tac). Risk factors were analyzed in 629 (92.2%) patients having at least 2 BKV measurements until month 12 posttransplant. Univariate analysis associated CsA-MPA with lower rates of viremia than Tac-MPA at month 6 (10.6% vs. 16.3%, p = 0.048) and 12 (4.8% vs. 12.1%, p = 0.004) and lower plasma BKV loads at month 12 (3.9 vs. 5.1 log_10_ copies/mL; p = 0.028). In multivariate models, CsA-MPA remained associated with less viremia than Tac-MPA at month 6 (OR 0.60; 95% CI 0.36–0.99) and month 12 (OR 0.33; 95% CI 0.16–0.68). Viremia at month 6 was also independently associated with higher steroid exposure until month 3 (OR 1.19 per 1 g), and with male gender (OR 2.49) and recipient age (OR 1.14 per 10 years) at month 12. The data suggest a dynamic risk factor evolution of BKV viremia consisting of higher corticosteroids until month 3, Tac-MPA compared to CsA-MPA at month 6 and Tac-MPA, older age, male gender at month 12 posttransplant.

## Introduction

In the last decade, polyomavirus BK-associated nephropathy (PyVAN) has emerged as significant cause of premature kidney transplant (KT) failure in many transplant centers around the world 1–3. PyVAN rates range from 1% to 10%, and progressive graft failure is seen in more than half of the cases 4–6. In recent analyses of large US databases covering approximately 40 000 KT patients during the period 2003–2006, treatment for BKV was reported in 6.6% during the first 5 years posttransplant and the adjusted risk of graft loss was at least twofold higher compared to unaffected patients 7,8. In the absence of specific antiviral therapy, current treatment relies on reducing immunosuppression using rising plasma BKV loads as a surrogate marker of disease 9–12. This approach can result in good clinical outcomes when performed early posttransplant 13,14. Accordingly, plasma BKV loads are currently recommended for screening and monitoring KT patients with presumptive and proven PyVAN 5,15,16. Despite growing consensus about screening, the risk factors for BKV viremia and nephropathy are not well defined 16. Most likely, nonmodifiable donor and recipient determinants synergize with potentially modulating factors such as immunosuppression 17. In the face of the unchanged seroepidemiology of BKV infection 9,18, increasing use of tacrolimus (Tac) compared to cyclosporine (CsA) has been discussed as a potential factor 19. However, while some studies reported a higher risk of BKV viruria, viremia and/or nephropathy in Tac-treated patients compared to CsA-treated patients 2,20, other studies were unable to identify such relation 11,21. To investigate the impact of the calcineurin inhibitor (CNI) directly, we examined BKV viruria and viremia in more than 600 *de novo* kidney transplant patients randomized 1:1 to Tac or CsA as part of the Diabetes Incidence after REnal Transplantation: Cyclosporine C2 monitoring versus Tacrolimus (DIRECT) study 22.

## Methods

### Patients

The DIRECT study is a prospective 6-month, open-label multicenter study with a follow-up visit at month 12 randomizing *de novo* KT patients to CsA or Tac. The study methodology has been described elsewhere 22. In brief, *de novo* renal transplant recipients aged 18–70 years (deceased, living-related or living-unrelated donor) were randomized 1:1 to CsA or Tac. Randomization was automated and investigators were notified via an interactive voice response system. The coprimary endpoints of this study were new onset diabetes or impaired fasting glucose, and biopsy-proven acute rejection, graft loss or death 22, and BKV replication was a secondary endpoint (see NCT00171496 at http://ClinicalTrials.gov). The study was performed in 59 transplant centers in 15 countries during October 2003 to March 2005 (see the Appendix). CsA (Neoral®, Novartis Pharma AG, Basel, Switzerland) dose was adjusted targeting C2 ranges: 1400–1800 ng/mL during month 1, 1200–1600 ng/mL during months 2–3 and 800–1200 ng/mL during months 4–6. Tac (Prograf®, Astellas Pharma, Tokyo, Japan) dosing was based on C0 targets: 10–15 ng/mL during months 1–3 and 5–10 ng/mL during months 4–6. All patients received mycophenolic acid (MPA) in the form of mycophenolate mofetil (MMF, Cellcept®, Roche Pharmaceuticals, Basel, Switzerland) or enteric-coated mycophenolate sodium (EC-MPS, myfortic®, Novartis Pharma AG) administered according to local practice, with corticosteroids (intravenous methylprednisolone 500 mg followed by oral prednisone tapered from 100–200 mg/day on day 1 to 5–10 mg/day from month 3 onward). Induction therapy consisted of two 20 mg doses of basiliximab (Simulect®, Novartis Pharma AG) given on days 0 and 4.

### Virological analysis

Collection of urine and EDTA blood samples was scheduled at baseline (i.e. pretransplantation or on the day of transplantation) and at months 1, 2, 3, 6 and 12. All samples were frozen at −20°C until analyzed by a quantitative real-time polymerase chain reaction 23 in the Division Infection Diagnostics, University of Basel (STS217 ISO/IEC-17025). BKV viruria was defined as detecting BKV DNA above a diagnostic threshold of 2500 copies/mL, high-level BKV viruria as urine DNA loads of >7 log_10_ copies/mL 5. BKV viremia was defined as plasma BKV loads above the lower diagnostic limit of detection of 1000 copies/mL, high-level BKV viremia as plasma BKV loads of >4 log_10_ copies/mL 5.

### Statistical analysis

Kaplan–Meier analyses were applied to determine cumulative incidences omitting patients with detectable BKV at baseline. Standard summary statistics were determined for numerical results. Missing samples were not imputed and not included in the analyses. In univariate analyses, we investigated potential determinants of BKV replication including age, gender, race, preexisting diabetes, HLA-mismatches, cold ischemia time, delayed graft function, donor status (living versus deceased), type of dialysis prior to transplantation, CMV status of donor and recipient, recipients’ hepatitis C virus (HCV) status and CNI. Multivariate logistic regression modeling was performed to investigate risk factors of BKV viruria and viremia at months 6 and 12. Odds ratios (OR) were calculated for 10 years of age and for 1 g of cumulative steroid dose, respectively. Cumulative steroid doses >10 g were censored at 10 g to avoid undue influence of exceptional outliers. Binary variables were used for CNI type (CsA vs. Tac), gender (male vs. female), race (white vs. nonwhite), history of diabetes mellitus (yes vs. no), sum of HLA mismatches at loci A, B and DR (>4 vs. ≤ 4) and delayed graft function (yes vs. no). In sensitivity analyses, BKV samples were omitted from analysis if they were acquired after discontinuation of the study medication. All p-values were two-tailed and considered significant at <0.05. Analyses were performed using SAS statistical software version 8.2 (SAS Institute, Cary, NC, USA).

## Results

A total of 3213 urine and 3531 plasma samples were obtained from 682 patients at the scheduled time points for BKV DNA testing. No posttransplant sample had been collected in 39 patients, and only one sample in 14 patients, together 53 patients excluded from further analysis. Thus, the BKV study population consisted of 629 kidney transplant recipients (92.2%) for whom a total of 3156 urine (98.2%) and 3465 plasma samples (98.1%) were obtained from at least two visits between months 1 and 6 posttransplant.

**Figure 1:**
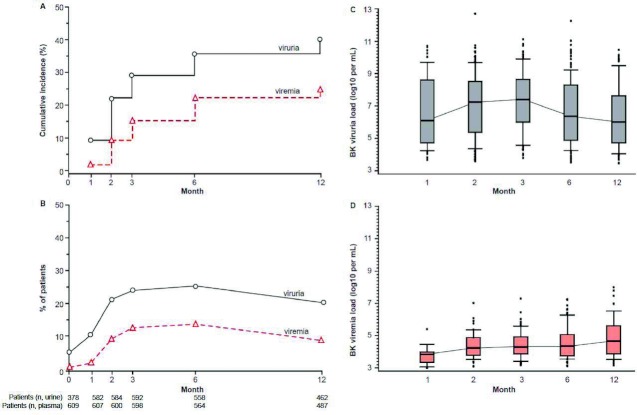
BKV viruria and viremia after kidney transplantation (A) Cumulative new-onset BKV replication posttransplant; (B) point prevalence at the times of testing (patient sample number below); (C) viral load in new-onset BKV viruria posttransplant; (D) viral load in new-onset BKV viremia postransplant

At baseline, BKV viruria was detected in 19 (5.0%) of 378 patients with residual urine production. None of these developed viremia, and only 8 remained viruric posttransplant at a low level of less than 5 log_10_ copies/mL. Baseline BKV viremia was found in 3 (0.5%) of 609 patients, but none had detectable viruria or viremia posttransplant. Kaplan–Meier estimates showed that the incidence of new onset BKV viruria and viremia at month 12 increased to 39.5% (95% CI 35.4%, 43.5%) and 23.9% (95% CI 20.4%, 27.3%), respectively (Figure 1A). Comparing different time points posttransplant, the highest rates of viruria and viremia were observed at month 6 (25.4% and 13.7%, respectively) which then decreased at month 12 (20.3% and 8.6%, respectively) (Figure 1B). Median urine BKV loads increased from 6.1 log_10_ copies/mL at month 1 to 7.4 log_10_ copies/mL at month 3 before declining to 6.0 log_10_ copies/mL at month 12 (Figure 1C). At that time point, one fourth of the samples (75th percentile) had very high urine viral loads above 8 log_10_ copies/mL. Plasma BKV loads increased from a median 3.8 log_10_ copies/mL at month 1 to 4.7 log_10_ copies/mL at month 12 (Figure 1D). Biopsy-proven acute rejection episodes were more frequent in patients with BKV viremia at month 6 (13.0% vs. 6.1%, p = 0.030) while no statistically significant association was found for viruria. The estimated glomerular filtration rate was not different for patients with or without viruria, but at month 12, viremic patients had a significantly impaired function compared to those without viremia (median GFR 60.4 mL/min [25th percentile 45.6, 75th percentile 78.2] vs. 65.7 mL/min [25th percentile 53.1, 75th percentile 83.5]; p = 0.032).

**Figure 2:**
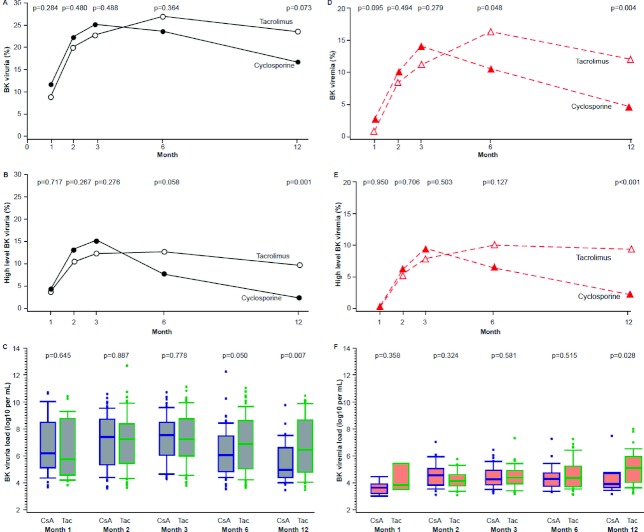
BKV viruria and viremia rates according to the treatment arm (A) BKV viruria; (B) BKV viruria above 7 log_10_ geq/mL (high-level viruria); (C) urine BKV loads in viruric patients; (D) BKV viremia; (E) BKV viremia above 4 log_10_ geq/mL (high-level viremia); (F) plasma BKV loads in viremic patients.

Patients randomized to either CNI arm were found to have similar baseline characteristics, including recipient male gender, white race, mean age, history of diabetes, delayed graft function, living donor and mean HLA mismatch (Table 1A). BKV viruria rates increased up to month 3 without significant differences between CsA- and Tac-randomized patients, but there was a trend toward less viruria among CsA-treated patients at month 12 (Figure 2A). At month 6, fewer patients in the CsA-treatment arm had high-level viruria of >7 log_10_ copies/mL compared to Tac-treated patients (p = 0.058) reaching statistically significance at month 12 (p = 0.001) (Figure 2B). Of note, median urine BKV loads were sevenfold lower (0.8 log_10_ copies/mL) in CsA- than in Tac-randomized patients at month 6 (p = 0.050) and approximately 30-fold lower (1.5 log_10_ copies/mL) at month 12 (p = 0.007; Figure 2C).

**Table 1A:** Demographic and baseline determinants in patients with or without BK viruria at months 6 and 12

	Month 6				Month 12			
		
Viruria	N	n+ (%)	n− (%)	p	N	n+ (%)	n− (%)	p
Total	558	142 (25.4%)	416 (74.6%)		462	94 (20.3%)	368 (79.7%)	
Recipient age				0.552				0.253
<40 years	183	47 (25.7%)	136 (74.3%)		156	25 (16.0%)	131 (84.0%)	
40–54 years	211	58 (27.5%)	153 (72.5%)		179	41 (22.9%)	138 (77.1%)	
≥ 55 years	164	37 (22.6%)	127 (77.4%)		127	28 (22.0%)	99 (78.0%)	
Gender				0.965				0.747
Male	382	97 (25.4%)	285 (74.6%)		323	67 (20.7%)	256 (79.3%)	
Female	176	45 (25.6%)	131 (74.4%)		139	27 (19.4%)	112 (80.6%)	
Race				0.811				0.836
White	468	120 (25.6%)	348 (74.4%)		395	81 (20.5%)	314 (79.5%)	
Nonwhite	90	22 (24.4%)	68 (75.6%)		67	13 (19.4%)	54 (80.6%)	
History of DM				0.760				0.704
Yes	87	21 (24.1%)	66 (75.9%)		68	15 (22.1%)	53 (77.9%)	
No	471	121 (25.7%)	350 (74.3%)		394	79 (20.1%)	315 (79.9%)	
HLA mismatches				0.964				0.631
0	23	6 (26.1%)	17 (73.9%)		18	4 (22.2%)	14 (77.8%)	
1–3	280	70 (25.0%)	210 (75.0%)		232	51 (22.0%)	181 (78.0%)	
4–6	254	66 (26.0%)	188 (74.0%)		212	39 (18.4%)	173 (81.6%)	
DGF				0.883				0.429
Yes	96	25 (26.0%)	71 (74.0%)		76	18 (23.7%)	58 (76.3%)	
No	462	117 (25.3%)	345 (74.7%)		386	76 (19.7%)	310 (80.3%)	
Donor status				0.095				0.051
Living	185	39 (21.1%)	146 (78.9%)		152	23 (15.1%)	129 (84.9%)	
Deceased	373	103 (27.6%)	270 (72.4%)		310	71 (22.9%)	239 (77.1%)	
Dialysis				0.738				0.820
None	57	15 (26.3%)	42 (73.7%)		47	11 (23.4%)	36 (76.6%)	
Hemodialysis	406	100 (24.6%)	306 (75.4%)		335	66 (19.7%)	269 (80.3%)	
Peritoneal	95	27 (28.4%)	68 (71.6%)		80	17 (21.3%)	63 (78.8%)	
CMV D/R				0.857				0.661
Neg./neg.	100	29 (29.0%)	71 (71.0%)		78	16 (20.5%)	62 (79.5%)	
Neg./pos.	99	26 (26.3%)	73 (73.7%)		90	22 (24.4%)	68 (75.6%)	
Pos./neg.	70	19 (27.1%)	51 (72.9%)		57	9 (15.8%)	48 (84.2%)	
Pos./pos.	248	61 (24.6%)	187 (75.4%)		201	42 (20.9%)	159 (79.1%)	
HCV D/R				0.704				0.881
Neg./neg.	543	138 (25.4%)	405 (74.6%)		450	91 (20.2%)	359 (79.8%)	
Neg./pos.	11	3 (27.3%)	8 (72.7%)		10	2 (20.0%)	8 (80.0%)	
Pos./neg.	0	0	0		0	0	0	
Pos./pos.	2	0	2 (100.0%)		1	0	1 (100.0%)	
Cold ischemia time				0.168				0.062
N		141	411			93	364	
Median (h)		14.3	12.0			15.0	12.0	
IQR		(0, 35.5)	(0, 30.0)			(0.3, 35.5)	(0, 29.2)	

**Table 1B:** Demographic and baseline determinants in patients with or without BK viruria at months 6 and 12

	Month 6				Month 12			
		
Viruria	N	n+ (%)	n− (%)	p	N	n+ (%)	n− (%)	p
Total	564	77 (13.7%)	487 (86.3%)		487	42 (8.6%)	445 (91.4%)	
Recipient age				0.720				0.104
<40 years	186	23 (12.4%)	163 (87.6%)		161	10 (6.2%)	151 (93.8%)	
40–54 years	212	32 (15.1%)	180 (84.9%)		185	14 (7.6%)	171 (92.4%)	
≥ 55 years	166	22 (13.3%)	144 (86.7%)		141	18 (12.8%)	123 (87.2%)	
Gender				0.937				0.052
Male	386	53 (13.7%)	333 (86.3%)		342	35 (10.2%)	307 (89.8%)	
Female	178	24 (13.5%)	154 (86.5%)		145	7 (4.8%)	138 (95.2%)	
Race				0.633				0.749
White	472	63 (13.3%)	409 (86.7%)		409	36 (8.8%)	373 (91.2%)	
Nonwhite	92	14 (15.2%)	78 (84.8%)		78	6 (7.7%)	72 (92.3%)	
								
History of DM				0.732				0.230
Yes	88	11 (12.5%)	77 (87.5%)		78	4 (5.1%)	74 (94.9%)	
No	476	66 (13.9%)	410 (86.1%)		409	38 (9.3%)	371 (90.7%)	
HLA mismatches				0.983				0.950
0	24	3 (12.5%)	21 (87.5%)		19	2 (10.5%)	17 (89.5%)	
1–3	279	38 (13.6%)	241 (86.4%)		242	21 (8.7%)	221 (91.3%)	
4–6	260	36 (13.8%)	224 (86.2%)		226	19 (8.4%)	207 (91.6%)	
DGF				0.292				0.718
Yes	97	10 (10.3%)	87 (89.7%)		83	8 (9.6%)	75 (90.4%)	
No	467	67 (14.3%)	400 (85.7%)		404	34 (8.4%)	370 (91.6%)	
Donor status				0.918				0.192
Living	186	25 (13.4%)	161 (86.6%)		160	10 (6.3%)	150 (93.8%)	
Deceased	378	52 (13.8%)	326 (86.2%)		327	32 (9.8%)	295 (90.2%)	
Dialysis				0.286				0.993
None	59	12 (20.3%)	47 (79.7%)		49	4 (8.2%)	45 (91.8%)	
Hemodialysis	410	53 (12.9%)	357 (87.1%)		357	31 (8.7%)	326 (91.3%)	
Peritoneal	95	12 (12.6%)	83 (87.4%)		81	7 (8.6%)	74 (91.4%)	
CMV D/R				0.535				0.232
Neg./neg.	102	18 (17.6%)	84 (82.4%)		85	7 (8.2%)	78 (91.8%)	
Neg./pos.	102	15 (14.7%)	87 (85.3%)		93	13 (14.0%)	80 (86.0%)	
Pos./neg.	71	7 (9.9%)	64 (90.1%)		64	6 (9.4%)	58 (90.6%)	
Pos./pos.	249	34 (13.7%)	215 (86.3%)		209	14 (6.7%)	195 (93.3%)	
HCV D/R				0.511				0.500
Neg./neg.	547	73 (13.3%)	474 (86.7%)		474	40 (8.4%)	434 (91.6%)	
Neg./pos.	13	3 (22.1%)	10 (76.9%)		11	2 (18.2%)	9 (81.8%)	
Pos./neg.	0	0	0		0	0	0	
Pos./pos.	2	0	2 (100.0%)		1	0	1 (100.0%)	
								
Cold ischemia time				0.845				0.965
N		76	482			41	441	
Median (h)		12.0	12.2			11.6	12.4	
IQR		(0, 35.5)	(0, 30.0)			(0, 24.0)	(0, 35.5)	

N = total number of patients within category; n+ = number of patients with viruria; n− = number of patients without viruria; DM = diabetes mellitus; HLA = human leukocyte antigen; DGF = delayed graft function; CMV = cytomegalovirus serology; HCV = hepatitis C; D/R = donor / recipient; neg. = negative; pos. = positive.

p-values from chi-square tests, for cold ischemia time from Wilcoxon rank-sum tests.

BKV viremia rates increased in both treatment arms over the first 3 months, but then diverged as patients in the CsA-arm had a lower rate of viremia compared to patients in the Tac-arm, both at month 6 (10.6% vs. 16.3%, p = 0.048) and at month 12 (4.8% vs. 12.1%, p = 0.004; Figure 2D). The on-treatment analysis revealed no significant differences in the incidence rates compared to the results presented above, e.g. BKV viremia at months 6 and 12 was 10.9% and 4.4% for the CsA- and 15.3% and 11.7% Tac-arm, respectively. The rate of high-level viremia of more than 10 000 copies/mL (4 log_10_) was lower in patients randomized to the CsA- than to Tac-arm at month 12 (2.2% vs. 9.4%, p<0.001; Figure 2E). Moreover, median plasma BKV loads were 15-fold (1.2 log_10_/mL) lower in CsA-MPA than in Tac-MPA treated patients (p = 0.028; Figure 2F).

Other potential determinants of BKV replication were not significantly associated with BKV viruria or BKV viremia, but we noted a trend toward a higher rate of viremia at month 12 for male patients (male: 10.2%, female 4.8%, p = 0.052) and for higher rates of viruria and viremia in deceased donors (Table 1A).

**Figure 3:**
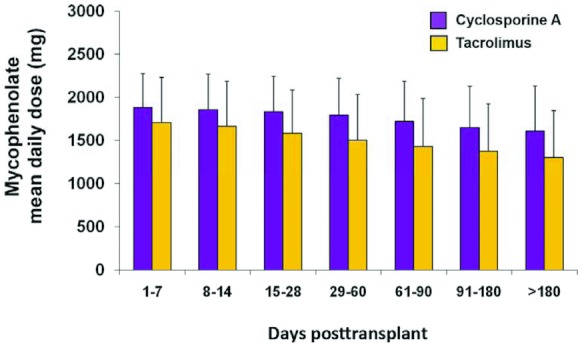
Mycophenolate dosing over time posttransplant by calcineurin inhibitor.

Patients on CsA reached the C2 target range on average by day 15. Patients with viruria at month 6 had slightly lower CsA C2 values at month 3 (median 1053; interquartile range [IQR] 825, 1255) than those without viruria (median 1173; IQR 900, 1500; p = 0.041). Similarly, CsA C2 values at month 6 were lower in viruric patients (median 833, IQR 610, 1016) compared to nonviruric patients (median 934, IQR 697, 1204, p = 0.025). Patients on Tac reached the C0 target range on average by day 11. An association between Tac trough levels and the occurrence of viruria or viremia could not be identified (all p-values >0.05). Since MPA exposure as measured by AUC was not determined in this study, MPA dosing was analyzed. MPA dosing decreased posttransplant in both treatment arms, but the mean dose of MPA was higher in CsA- than in Tac-treated patients at all time points in line with current clinical practice to accommodate lower exposure in CsA-treated patients (p-values < 0.001; Figure 3). MPA-dosing was not different between patients with or without BKV viruria or viremia at month 6 (all p-values > 0.10). To exclude undefined effects of the different CNI–MPA interaction, MPA-dosing was examined separately in patients of either treatment arm. There was no significant difference of MPA dosing in months 4–6 among Tac-MPA treated patients with or without viruria or viremia at month 6.

Examining the role of steroids, we found no association with BKV viruria, but BKV viremia was significantly associated with a higher cumulative steroid exposure until month 1 having a median of 1470 mg (IQR 995 mg, 1808 mg) compared to 1250 mg (IQR 870 mg, 1655 mg) for patients without viremia (p = 0.031; Table 2). This difference persisted up to month 6 suggesting that corticosteroid exposure was an important modulator of the risk of BKV replication early posttransplant.

**Table 2:** Cumulative dose of corticosteroids in patients with or without BK viruria and viremia at month 6

		Viruria at month 6			Viremia at month 6		
			
Period^1^		Viruria	No viruria	p	Viremia	No viremia	p
≤Month 1	Median	1241	1274	0.572	1470	1250	0.031
	(Q1, Q3)	(930, 1689)	(888, 1678)		(995, 1808)	(870, 1655)	
≤Month 2	Median	1831	1795	0.409	1924	1770	0.017
	(Q1, Q3)	(1370, 2318)	(1330, 2205)		(1476, 2409)	(1313, 2203)	
≤Month 3	Median	2161	2112	0.403	2315	2100	0.013
	(Q1, Q3)	(1625, 2791)	(1616, 2642)		(1815, 3009)	(1606, 2643)	
≤Month 6	Median	3094	2898	0.404	3158	2897	0.021
	(Q1, Q3)	(2180, 3785)	(2160, 3593)		(2549, 4217)	(2145, 3585)	

1 Cumulative dose in mg determined from transplantation up to end of the respective month.

Q1 = first quartile; Q3 = third quartile.

p-values from Wilcoxon rank-sum tests.

In the multivariate logistic regression model, BKV viruria was not significantly associated with any of the variables (Table 3). Investigating high-level viruria, however, randomization to CsA-MPA treatment significantly decreased the risk compared to Tac-MPA at month 6 (OR = 0.56, 95% CI 0.31, 0.99; p = 0.047) and month 12 (OR = 0.21, 95% CI 0.08, 0.57; p = 0.002). The cumulative steroid dose was still significant at month 6 (per 1 g higher: OR = 1.26), but not at month 12 (Table 3). For BKV viremia at month 6, CsA-MPA remained an independent factor decreasing risk compared to Tac-MPA (OR = 0.60, p = 0.044), while higher cumulative steroid dose increased the risk (per 1 g higher: OR = 1.19 per 1 g; p = 0.017). For BKV viremia at month 12, CsA-MPA was associated with decreased risk (OR of 0.33; p = 0.003). BKV viremia was independently associated with male gender (OR = 2.49; p = 0.038) and increasing age (OR = 1.41 per 10 years; p = 0.013; Table 3). For high-level viremia at month 12, CsA-MPA remained significant (OR = 0.19; p = 0.001), whereas age, gender or steroids were not.

**Table 3:** Results from logistic regression analyses for occurrence of BK viruria and viremia at months 6 and 12 posttransplant dependent on the CNI type and other potential risk factors

	Viruria (n = 558)			Viremia (n = 564)		
		
Month 6	OR	95% CI	p-Value	OR	95% CI	p-Value
CNI (CsA vs. Tac)	0.85	(0.57, 1.24)	0.394	0.60	(0.36, 0.99)	0.044
Age^a^	0.99	(0.85, 1.16)	0.928	1.14	(0.94, 1.40)	0.187
Gender (male vs. female)	1.00	(0.66, 1.51)	0.992	1.03	(0.61, 1.74)	0.920
Race (white vs. nonwhite)	1.04	(0.60, 1.81)	0.892	0.69	(0.35, 1.34)	0.272
History of DM (yes vs. no)	1.10	(0.63, 1.92)	0.724	1.32	(0.64, 2.72)	0.449
HLA mismatches (>4 vs. < = 4)	0.88	(0.55, 1.40)	0.581	1.21	(0.66, 2.21)	0.544
DGF (yes vs. no)	0.98	(0.59, 1.64)	0.947	1.62	(0.79, 3.32)	0.192
Cumulative steroid dose^2^	1.09	(0.96, 1.24)	0.197	1.19	(1.03, 1.38)	0.017

	Viruria (n = 462)			Viremia (n = 487)		
		
Month 12	OR	95% CI	p-Value	OR	95% CI	p-Value
CNI (CsA vs. Tac)	0.64	(0.40, 1.03)	0.065	0.33	(0.16, 0.68)	0.003
Age^1^	1.19	(0.98, 1.44)	0.076	1.41	(1.07, 1.84)	0.013
Gender (male vs. female)	1.12	(0.67, 1.86)	0.661	2.49	(1.05, 5.89)	0.038
Race (white vs. nonwhite)	0.91	(0.46, 1.81)	0.791	0.75	(0.29, 1.96)	0.556
History of DM (yes vs. no)	1.00	(0.52, 1.92)	0.994	2.52	(0.83, 7.65)	0.103
HLA mismatches (>4 vs. < = 4)	1.16	(0.65, 2.05)	0.618	1.49	(0.64, 3.46)	0.349
DGF (yes vs. no)	0.84	(0.46, 1.54)	0.579	0.80	(0.34, 1.86)	0.602
Cumulative steroid dose^2^	1.08	(0.93, 1.25)	0.322	1.09	(0.88, 1.35)	0.424

1 Odds ratio represents an increment of 10 years.

2 Cumulative steroids dose up to month 3, odds ratio represents an increment of 1 g.

OR = odds ratio; CI = confidence interval; CNI = calcineurin inhibitor; CsA = cyclosporine; Tac = tacrolimus; DM = diabetes mellitus; HLA = human leukocyte antigen; DGF = delayed graft function defined as a decrease in serum creatinine ≤ 20% or the need for at least one dialysis in the first 3 days posttransplantation.

## Discussion

This prospective, randomized, multicenter study provides the largest systematic analysis of BKV viruria and viremia after kidney transplantation using a predefined protocol of immunosuppression. The results demonstrate that reactivation of BKV replication is common with BKV viruria reaching a cumulative incidence of 39.5% (95% CI 35.4%, 43.5%) by 12 months posttransplant. One-fourth of KT patients developed high-level viruria of >7 log_10_ copies/mL, a molecular equivalent of urinary decoy cell shedding, as well as viremia, both biomarkers of an increasing risk of progression to PyVAN 9,24. Plasma BKV loads >4 log_10_ copies/mL were observed in 16% of KT patients, thereby fulfilling the working definition of presumptive PyVAN for which judicious reduction of immunosuppression is currently recommended 15,16. The rates are in line with a smaller prospective study of 78 patients by Hirsch et al. reporting Kaplan–Meier estimates of high-level viruria (decoy cells) of 30% (95% CI 20–40%) and viremia of 13% (95% CI 5–21%) 9, as well as with results by Brennan et al. 2005 11 and Ginveri et al. 2007 13.

Randomizing more than 600 patients 1:1 to either CsA or Tac on a common backbone of basiliximab induction, MPA and prednisone provided an unprecedented large sample size associating CsA-MPA with a significantly lower rate of viremia than Tac-MPA, both at month 6 (10.6% vs. 16.3%, p = 0.048) and at month 12 (4.8% vs. 12.1%, p = 0.004). Furthermore, median plasma BKV loads were 10-fold higher at month 12 in Tac-MPA compared to CsA-MPA treated patients and high-level BKV viremia of >4 log_10_ copies/mL was significantly more frequent. In the first 3 months, however, the rate of BKV replication did not significantly differ between patients randomized to CsA or Tac suggesting that differences between the CNIs did not play out early, but during the second half of the first year posttransplant, when most cases of PyVAN had been previously diagnosed 1,4,6. Multivariate analysis confirmed the reduced risk of CsA-MPA treated patients for BKV viremia at months 6 and 12 compared to Tac-MPA, and identified higher steroid exposure in the first 3 months as an independent cofactor for high-level viruria and viremia. This association is of interest since it provides a rationale for the early onset of BKV replication, independent of the choice of CNI. Indeed, pulse-steroids had been identified as independent risk factor for high-level viruria (decoy cells), viremia and PyVAN 9. Dadhania and colleagues reported that steroid maintenance therapy was associated with BKV replication 25. The BKV-promoting effect of steroids likely is the result of both, activating BKV early gene expression via glucocorticoid response elements in the viral noncoding control region 26 and its immunosuppressive effect. This synergy of virus activation and immunity inactivation is also documented in the poor outcome of polyomavirus-associated nephropathy almost a decade ago when the disease was erroneously treated as acute cellular rejection 1,2. The results of our study further suggest that following protocol-driven corticosteroid dose tapering from month 3 onward, the choice CNI exerted a greater influence on BKV replication rates. Of note, male gender and older recipient age were identified as independent risk factors for BKV viremia at month 12. Both patient determinants have been reported previously as being associated with PyVAN in some single-center studies 4,27 as well as in the large UNOS/OPTN registry analyses 7,8.

Brennan et al. 11 randomized 200 KT patients in a ratio of 2:1 to either Tac or CsA that was combined with either azathioprine or MPA 11. While the CNI *per se* was not found to influence the overall rates of BKV viruria and viremia in that study, there was a trend for an increased rate of sustained viremia in Tac- versus CsA-treated patients (p = 0.10). Similar to our results, BKV viruria was more frequent among Tac-MPA versus CsA-MPA treated patients (46% vs. 13%; p = 0.005). The rates of viremia in Tac-MPA versus CsA-MPA treated patients were similar to our study (13% vs. 4%), but without reaching statistical significance which may reflect their smaller sample size of 88 patients only in these comparator arms 11 compared to 629 patients reported here.

The difference between both Tac-MPA and CsA-MPA regarding BKV has previously been attributed to the potential influence of a higher overall immunosuppressive burden of Tac-containing regimens. In this study, CsA was targeted according C2 monitoring providing optimal exposure early posttransplant reaching median concentrations of 885 ng/mL at month 6, while tacrolimus was standard-dosed with predefined tapering reaching trough levels of 8 ng/mL at month 6. Under these conditions, the primary efficacy endpoints were found to be comparable including biopsy-proven acute rejection 22. The significant association of BKV viremia with patients randomized to Tac may also be influenced by MPA exposure, but frequent and possibly more precise pharmacokinetic measurements were not performed 28. On the other hand, representative time points posttransplant are not defined for comprehensive AUC measurements. This particularly concerns the question when to expect effects on BKV viremia, since pharmacokinetic measurements only provide data as a point prevalence that are not necessarily representative of concurrent, cumulative or subsequent effects. In our study, however, MPA dosing, decreased posttransplant and was, at all time points, significantly lower in patients randomized to Tac compared to CsA, in agreement with common routine practice to reduce differences in exposure (Figure 3). Of note, we found no difference in MPA doses between viruric or nonviruric, or between viremic and nonviremic patients within either CNI treatment group at month 6. Although screening was not yet widely recommended and practiced during the study period 15,29, we cannot exclude that treatment for BKV viremia might have occurred and thereby shortened viremia duration in some patients. However, even with proactive reduction of immunosuppression, the median duration of BKV viremia is long ranging from 2.9 to 8.8 months 14,30. Given this long duration, we consider it unlikely that potential screening and treatment of some centers might have significantly changed the results.

Agent-specific mechanisms may also play a role in the different clinical adverse event profile of Tac and CsA as evidenced by changes in glucose or lipid metabolism 22. Although Tac and CsA both inhibit the calcineurin phosphatase required for interleukin-2 expression in T-lymphocytes subsequent to T cell receptor activation, they have different molecular targets: CsA binds to cyclophilins while tacrolimus binds to FK-binding protein 12. Interestingly, *in vitro* studies indicate that CsA and MPA inhibit BKV replication 31–33, whereas Tac activates BKV replication via FK-binding protein 12 in primary human tubular epithelial cells 34. The “net state of immunosuppression” coined by Rubin and Fishman 35 may be used to also integrate net effects on virus replication by different drugs as well as quantitative and qualitative differences of virus-specific T cell repertoire in the individual transplant recipients 36. Clearly, at high doses of immunosuppressive drugs, BKV-inhibitory effects of CsA, mTOR inhibitors and MPA may not play out and immunosuppressive effects predominate. As dosing is lowered posttransplant, e.g. at 3 months onward, however, drug-specific differences may become apparent and BKV-activating effects as described for corticosteroids and Tac may increase the risk over drugs with BKV-inhibitory effects such as CsA, mTOR inhibitors and MPA, at an otherwise appropriate maintenance immunosuppression for a given patient–allograft combination. The role of differential direct activating and inhibiting viral effects of immunosuppressive drugs like tacrolimus versus cyclosporine, MPA and mTOR inhibitors are currently emerging, together with their differences in immunosuppressive action. The improved understanding direct drug mechanisms on infectious agents and the immune system will stimulate more specific clinical studies that allow to better evaluate the competing risks of rejection and infection in future personalized transplantation medicine.

In conclusion, CsA is associated with a significantly lower risk than Tac regarding BKV viremia at months 6 and 12 in *de novo* kidney transplant patients treated with basiliximab, MPA and steroids. Steroids appear as an independent risk factor for BKV viremia early posttransplant, whereas male gender and older age contribute later in the first year posttransplant, respectively. Together, these data support the hypothesis of a dynamic risk evolution across multiple factors including the choice of the CNI, thereby potentially influencing the screening after 6 months posttransplant and the management of patients at higher risk for BKV viremia and progression to nephropathy.

## Abbreviations

BKV: BK virus
CsA: cyclosporine-A
CNI: calcineurin inhibitor
DIRECT: Diabetes Incidence after REnal Transplantation Cyclosporine C2 monitoring versus Tacrolimus
GFR: glomerular filtration rate; HLA, human leukocyte antigen
HCV: hepatitis C virus
KT: kidney transplant
MMF: myophenolate mofetil
MPA: mycophenolic acid; PyVAN, polyomavirus-associated nephropathy
Tac: tacrolimus
